# How Children Feel Matters: Teacher–Student Relationship as an Indirect Role Between Interpersonal Trust and Social Adjustment

**DOI:** 10.3389/fpsyg.2020.581235

**Published:** 2021-01-18

**Authors:** Yan Dong, Hongfei Wang, Fang Luan, Zheneng Li, Li Cheng

**Affiliations:** ^1^Department of Psychology, Renmin University of China, Beijing, China; ^2^Faculty of Education, Beijing Normal University, Beijing, China; ^3^Developmental and Educational Research Center for Children’s Creativity, Faculty of Education, Beijing Normal University, Beijing, China

**Keywords:** interpersonal trust, teacher-student relationship, social adjustment, indirect role, pupils

## Abstract

Previous studies have demonstrated positive correlations between children’s interpersonal trust and social adjustment. However, the psychological mechanism underlying this effect is still unclear. The current study tested the indirect roles of teacher–student relationships from both students’ and teachers’ perspectives in a Chinese context. In total, 709 pupils from grade three to grade five, and their 17 head teachers from a Chinese public primary school participated in this study. The Children’s Generalized Trust Beliefs Scale, Social Adjustment Scale for Children and Adolescents, and Teacher–Student Relationship Questionnaire were used in this study. All these variables were correlated with each other. Structural equation models showed that the interpersonal trust indirectly influenced social adjustment through the teacher–student relationship from students’ perspectives, while the teacher–student relationship from teachers’ perspectives did not play an indirect role. These findings suggest that the teacher–student relationship perceived by students is more important for children’s social adjustment than that perceived by teachers. Both parents and teachers should pay more attention to developing children’s interpersonal trust, build better teacher–student relationships, and focus more on how children feel about the relationship.

## Introduction

Interpersonal trust has been proved to have positive influence on children’s social adjustment, which is beneficial to their individual development (Rotter, 1980; Betts and Rotenberg, 2007; Rotenberg et al., 2008; Betts et al., 2009, 2013; Wang and Fletcher, 2016). According to the norm of reciprocity in social exchange, a higher level of interpersonal trust positively contributes to better teacher–student relationships (Blau, 1964; Rotter, 1980), which may in turn facilitate children’s social adjustment (Pianta et al., 1995; Baker et al., 2008; Liu et al., 2015). However, the teacher–student relationships can be assessed by teachers or students, respectively (Rey et al., 2007). Whether the teacher–student relationships from students’ and teachers’ perspectives will play different indirect roles remains unclear.

Children’s ability to have friendly interactions with others in a social environment is crucial for their development. In the past decade, school psychologists not only focus on children’s academic skills, but also pay more attention to children’s social competence, such as social adjustment (McCoy et al., 2013; Yang et al., 2015; Abaied and Stanger, 2017). Social adjustment is defined as the interplay between the individuals and their social environment (Weissman, 1975). For children, some scholars have proposed that social adjustment in childhood consists of three elements, namely, self-concept, emotional well-being, and school enjoyment (Wang et al., 2016). Social adjustment in childhood has particular significance in both individual development and educational practice. For example, children with social adjustment difficulties exhibit more internalizing and externalizing problems (Bornstein et al., 2010).

Many intrapersonal factors related to children’s social adjustment have been explored in past years (Abaied and Stanger, 2017; Oliver and Pike, 2018). One of such factors is interpersonal trust, defined as “a generalized expectancy held by an individual that the word, promise, oral or written statement of another individual or group can be relied on” (Betts and Rotenberg, 2007). According to Rotenberg’s conceptual model of interpersonal trust, there are three bases of interpersonal trust: reliability, emotional, and honesty (Rotenberg et al., 2005a). This framework has suggested that interpersonal trust is conceptualized as having strong reciprocal qualities, notably with social interactions (Rotenberg et al., 2008). Scholars believe that the children’s interpersonal trust contributes to their social adjustment for two reasons. The first is that children with higher levels of interpersonal trust may develop better friendships with peers, which may in turn facilitate social adjustment (Betts and Rotenberg, 2007; Betts et al., 2013). The second is that children’s interpersonal trust is positively associated with honesty, academic achievement, and effective interpersonal problem solving, which may also promote children’s social adjustment (Rotenberg et al., 2005a). Numerous empirical studies have discovered that children’s interpersonal trust has a positive influence on social adjustment (Betts and Rotenberg, 2007; Rotenberg et al., 2008; Betts et al., 2009, 2013; Wang and Fletcher, 2016). However, the psychological mechanism underlying the effect of interpersonal trust on social adjustment remains unclear.

### Interpersonal Trust’s Indirect Effect on Social Adjustment

Erikson (1963) proposed that trust is formed during one’s early years, and will impact interpersonal functioning in his or her later life. People with higher levels of interpersonal trust tend to hold higher expectancy on other people’s words, promises, and statements, which may lead to better relationships between people (Rotter, 1980). Empirical studies have found that one’s high interpersonal trust level can contribute to interpersonal relationships (Matzler and Renzl, 2006; Rotenberg and Boulton, 2013). In the school environment, children’s interpersonal trust acts as the “glue” to form and maintain social relationships (Rotenberg et al., 2005a; Rotenberg, 2010; Betts et al., 2013). Previous studies mainly focused on the effect of children’s interpersonal trust on peer relationships (Betts et al., 2013; Rotenberg and Boulton, 2013); however, to our knowledge, little research has explored the effect of interpersonal trust on teacher–student relationships. For those children with higher levels of interpersonal trust, they may hold higher expectancy on their teachers’ words, promises, and statements (Rotter, 1980), which makes children more willing to interact with their teacher. According to the norm of reciprocity in social exchange (Blau, 1964), teachers will pay more attention to these children and make contact with them more frequently, resulting in better teacher–student relationships. Therefore, children’s interpersonal trust may contribute to teacher–student relationships.

A teacher plays a vital role in children’s school years (Pianta et al., 1995; Ettekal and Shi, 2020). On the basis of attachment theory, positive teacher–student relationships in childhood may provide children with security and enhance their adjustment to school (Bowlby, 1980). Empirical studies have demonstrated the influence of teacher–student relationships on children’s social adjustment (Pianta et al., 1995; Baker et al., 2008; Liu et al., 2015; Ang et al., 2020; Ettekal and Shi, 2020). Positive teacher–student relationships, featuring warmth, nurturance, and low negativity have been verified to exert positive effects on children’s school lives (Hughes et al., 2012), and enhance social adjustment in childhood (Pianta et al., 1995; Baker et al., 2008). Some other studies on young children have shown that teacher–student relationships positively impact children’s mental health, learning engagement, and academic performance, which are all beneficial to children’s social adjustment (Zee et al., 2013; Miller-Lewis et al., 2014; Bosman et al., 2018). Since teacher–student relationship is associated with children’s interpersonal trust and social adjustment, teacher–student relationship may play an indirect role between children’s interpersonal trust and social adjustment.

### The Teacher–Student Relationships From Different Perspectives

As suggested by the anchoring-and-adjustment heuristic (Gilovich et al., 2000; Epley, 2008), people tended to evaluate others’ mental states by starting with their own and then adjusting it, albeit insufficiently, to take others’ perspectives into account; this implied that neither students nor teachers may evaluate the relationship between them sufficiently. Since teacher–student relationship is the result of mutual interaction between teachers and students, it is worth noting that both teachers and students have their own perspectives on the teacher–student relationship. Some studies have measured this relationship from the perspective of students, that is, the students evaluated and reported on the relationship between the teachers and themselves (e.g., Hughes et al., 2012; Wang et al., 2013; Liu et al., 2015); while some other studies assessed the teacher–student relationship from teachers’ self-reports (e.g., Pianta et al., 1995; Hughes, 2011). A study on grade 3 to 6 students measured the teacher–student relationships from both perspectives, discovering that teachers’ perception of the teacher–student relationship better predicts teachers’ rated outcomes, whereas students’ perception of the relationship better predicts students’ rated outcomes (Rey et al., 2007). Another study has found that teacher and student reports of teacher–student relationships predict different academic outcomes, to be specific, students’ perceptions of the teacher–student relationship predict school belonging and math achievement, while teachers’ perceptions predict behavioral engagement (Hughes, 2011). On the whole, these findings and concerns indicate that perception of the teacher–student relationship may differ between teachers and students, and may have different effects on children’s social adjustment. Therefore, the indirect roles of the teacher–student relationship should be examined separately, in order to take the different perspectives of teachers and students into account.

### Teacher–Student Relationship in the Chinese Context

We focused on the teacher–student relationship in the Chinese context, which may be different from that of western contexts. Chinese culture highly emphasizes that students should respect their teachers, and Chinese students may attach great importance to the teacher–student relationship (Liu et al., 2015). For example, there is an ancient Chinese idiom “a teacher for a day is a father for a lifetime,” which means that students should respect the teacher as they respect their father. Similarly, some other East Asian countries (e.g., Japan, South Korea, India) also emphasize the importance of respecting teachers (Darling-Hammond and Cobb, 1996).

Besides, Chinese schools have larger class sizes than schools in America and European countries (Graue and Rauscher, 2009; Shen and Konstantopoulos, 2017). The class size required by the Chinese government is below 45, and most schools have about 40 students in each class. Teachers in large size classes may not give enough attention to every student in their classes. As a result, teachers and students in large size classes are more likely to evaluate and understand the teacher–student relationship differently.

Therefore, it is necessary to explore the different effects of the teacher–student relationships from both students’ and teachers’ perspectives in the Chinese context, which may provide implications for schools in similar cultural contexts (e.g., Japan, South Korea, India, Darling-Hammond and Cobb, 1996) and schools with large class sizes (e.g., schools in Africa, Foley and Masingila, 2014); however, few studies have focused on this issue.

### The Current Study

The current study aims to test the influence of interpersonal trust on social adjustment, and explore the psychological mechanism underlying this influence among grade 3–5 pupils (9–12 years old) in China. Among grade 3–5, peers start to show social preference for those students who have better relationships with teachers. This kind of social preference will also contribute to children’s social adjustment (Hughes et al., 2001). However, previous studies on primary school teacher–student relationships mainly focused on younger children, and research on middle grade pupils remains limited (Rotenberg et al., 2008; Hughes, 2011; Betts et al., 2013). The existing studies on teacher–student relationships among grade 3–5 students have found that the teacher–student relationship contributes to school adjustment and academic performance (Wang and Wang, 2006; Rey et al., 2007), while none of them explored the potential indirect effects of teacher–student relationship between interpersonal trust and social adjustment.

As previously mentioned, students and teachers in the Chinese context may evaluate and understand the teacher–student relationship differently. The current study aimed to evaluate the teacher–student relationships from both students’ and teachers’ perspectives in the Chinese context, contributing to a culture-specific understanding of the teacher–student relationship in similar cultural contexts (e.g., Japan, South Korea, India, Darling-Hammond and Cobb, 1996) and schools with large class sizes (e.g., schools in Africa, Foley and Masingila, 2014).

On the basis of the previous findings (Pianta et al., 1995; Betts and Rotenberg, 2007; Baker et al., 2008; Rotenberg et al., 2008; Betts et al., 2009, 2013; Liu et al., 2015; Wang and Fletcher, 2016), hypothesis 1 was proposed: *Among grade 3–5 students, students’ interpersonal trust, social adjustment, and the teacher–student relationship would be positively correlated with each other.*

Combining the previous findings and the discussed evidence, we speculate that students’ interpersonal trust has indirect effects on social adjustment through the teacher–student relationship. Moreover, the evaluation of the teacher–student relationship may differ between teachers and students, and may have different effects on children’s social adjustment. Therefore, hypothesis 2 was proposed: *Among grade 3–5 students, interpersonal trust would indirectly affect social adjustment through the teacher–student relationship, and the teacher–student relationships from students’ and teachers’ perspectives would play different indirect roles.*

## Materials and Methods

### Participants and Procedure

We used convenient sampling method to choose a mid-level local public primary school in Shanghai, China. We used a cluster sampling method to include 709 pupils in grades 3 to 5 (9–12 years old), and 17 head teachers (referring to the lead teachers of the classes, who were in charge of students’ school life) of these students in this school. Each head teacher was in charge of 42 students on average.

The students answered three online questionnaires, and the head teachers were instructed to answer the teachers’ section of the teacher–student relationship questionnaire to evaluate their relationships with *every* student in their classes. The surveys were non-anonymous. Students should write down their names and student number, and head teachers answered a separate questionnaire for each student in their class, so that the student and teacher questionnaires could be matched one by one. Finally, 592 pairs of effective questionnaires were matched; the other questionnaires failed to be matched, because some students did not complete all the questionnaires or write down their (real) names. Among the 592 pairs of efficient questionnaires, the demographic information of the students was shown in Table 1. All the head teachers were females, aged from 25 to 64 years (*M* = 41.94 ± 11.36), with an average 21 years’ teaching experience (*SD* = 11.96). The data collection took a month to complete (started in April 2017, and finished in May 2017). The University Ethics Review Board granted research approval. The head teachers gave informed consent at school; the student participants brought informed consent forms to their parents for review and potential signature, and returned informed consent forms within 3 days.

**TABLE 1:** Student participants’ demographic information.

	**Grade 3**	**Grade 4**	**Grade 5**	**In total**
In total	192	233	167	592
Male	102	125	81	308
Female	90	108	86	284
Mean age	9.59 ± 0.61	10.44 ± 0.52	11.49 ± 0.51	10.46 ± 0.92

### Measures

#### Interpersonal Trust

The Children’s Generalized Trust Beliefs (CGTB) Scale (Rotenberg et al., 2005b) was used to assess students’ interpersonal trust. This survey was answered by student participants. This scale measures three aspects of trust: *reliability* (which refers to promise or fulfillment of your word), *emotionality* (which refers to relying on others to avoid emotional harm, such as refraining from criticism or embarrassment), and *honesty* (which refers to speaking the truth, and engaging in benign or genuine behaviors instead of malicious or manipulative behaviors). Each aspect contains four target groups, *father*, *mother*, *teacher*, and *peer*, and each target group includes two items. The CGTB Scale has been widely used in both international and Chinese studies, with acceptable reliability and validity (e.g., Xu et al., 2013; Rotenberg et al., 2015; Petrocchi et al., 2017). Gong and Luo (2016) adapted this scale to its Chinese version and proved its reliability and validity. The items in this scale were selected to be ecologically representative: for example, (1) “Tingting’s mother said that if she cleans her room she can go to bed half-an-hour later. Tingting cleans her room. How likely is it that Tingting’s Mother will let Tingting go to bed half an-hour later?” (reliability aspect, mother target); (2) “Xiaoma tells her father that she is struggling with her schoolwork, but asks her father not to tell others about it. How likely is it that Xiaoma’s father will not tell others about it?” (emotionality aspect, father target); and (3) “Yaoyao asks Xiaoling to go to the cinema. Xiaoling says she cannot go because she feels tired. How likely is it that Xiaoling is tired?” (honesty aspect, peer target). Responses ranged from 1 (totally impossible) to 5 (totally possible) with higher scores indicative of higher levels of interpersonal trust. Previous studies showed that this scale was suitable for children aged 9–12 years (Rotenberg et al., 2015; Petrocchi et al., 2017). In this study, we reexamined the structure validity by a confirmatory factor analysis (CFA); the questionnaire exhibited an acceptable fit: χ^2^/*df* = 2.36, comparative fit index (CFI) = 0.92, Tucker–Lewis index (TLI) = 0.91, root mean square error of approximation (RMSEA) = 0.05, standardized root mean square residual (SRMR) = 0.04. As Hu and Bentler (1998, 1999) suggested, χ^2^/*df* value ≤3, CFI and TLI values ≥0.90–0.94, RMSEA values ≤0.06–0.08, and SRMR values ≤0.06–0.08, indicated an adequate fit. Cronbach’s alphas of the three aspects (reliability, emotionality, and honesty) and the whole questionnaire were 0.78, 0.75, 0.80, and 0.90.

#### Social Adjustment

Social Adjustment Scale for Children and Adolescents (SASCA) (Hu and Guo, 2013) was used to measure students’ social adjustment. This survey was answered by student participants. This scale includes 48 items and eight factors, namely (1) *learning autonomy* (e.g., “I try to find a better way to study.”), (2) *life independence* (e.g., “I wash my own clothes.”), (3) *environment satisfaction* (e.g., “My present class is very harmonious.”), (4) *friendly relationships* (e.g., “I have many friends.”), (5) *activity engagement* (e.g., “I am willing to take part in some voluntary labor.”), (6) *interpersonal coordination* (e.g., “I think it is normal for students to have different opinions on some issues.”), (7) *social identification* (e.g., “When there are problems among my classmates, I can help them get back together.”), and (8) *personal vitality* (e.g., “I am a lively and cheerful person.”). The SASCA has been used in previous Chinese studies, and proved to have good reliability and validity (e.g., Hu and Guo, 2013). Responses ranged from 1 (strongly disagree) to 5 (strongly agree), and higher scores were indicative of a higher level of social adjustment. In this study, the questionnaire exhibited acceptable structure validity, the fit χ^2^/*df* = 2.46, CFI = 0.90, TLI = 0.90, RMSEA = 0.05, SRMR = 0.05. Cronbach’s alphas of the eight factors from learning autonomy to personal vitality and the whole questionnaire were 0.82, 0.82, 0.74, 0.85, 0.79, 0.69, 0.81, 0.77, and 0.94, respectively.

#### Teacher–Student Relationship

Teacher–Student Relationship Questionnaire (Wang and Wang, 2006) was used in the current study. There are two versions of this questionnaire, one for teachers and one for students, assessing the relationship from both perspectives. Each version contains 28 items, with corresponding items for teachers and students. For example, the teacher item “the relationship between this student and I is close and warm,” corresponds to the student item “the relationship between my head teacher and I is close and warm.” The student participants answered the student version, and the head teachers were instructed to evaluate their relationship with every student in the class with the teacher version. This questionnaire measures three aspects of the teacher–student relationship, namely *proximity* (attitude and behavior of mutual identification, e.g., “the relationship between this student and I is close and warm”), *conflict* (cognitive, emotional, and behavioral inconformity, e.g., “I feel that my head teacher treats me unfairly”), and *reaction* (activeness in emotional and behavioral communication, e.g., “When I praise this student, he/she looks proud and radiant”). The teacher–student relationship questionnaire has been used in previous Chinese studies, and proved to have acceptable reliability and validity (e.g., Zou et al., 2007; Jin et al., 2012). Responses ranged from 1 (strongly disagree) to 5 (strongly agree), and 13 of the 28 items were reverse-scoring (item 2, 4, 6, 11, 13, 15, 16, 18, 20, 22–25). Higher scores were indicative of better teacher–student relationships. Conducting CFA, we found that the factor loadings of items 6, 8, 12, 14, 19, and 26 were relatively low (factor loadings <0.30, *p* > 0.05); therefore, these items were deleted. The subsequent CFA indicated that the questionnaire exhibited acceptable fits. The model fit of the student version was χ^2^/*df* = 2.67, CFI = 0.93, TLI = 0.92, RMSEA = 0.05, SRMR = 0.05. The model fit of the teacher version was χ^2^/*df* = 2.89, CFI = 0.91, TLI = 0.90, RMSEA = 0.08, SRMR = 0.06. Cronbach’s alphas of proximity, reaction, conflict, and the whole subscale of student version were 0.73, 0.73, 0.78, and 0.82, respectively; Cronbach’s alphas of proximity, reaction, conflict, and the whole subscale of teacher version were 0.76, 0.75, 0.82, and 0.87, respectively.

### Data Analysis

To assess for common method bias, the Harman single factor test was employed to test the questionnaires answered by students (Harris and Mossholder, 1996). All variables were subjected to exploratory factor analysis; results indicated that the single factor with the largest explanatory power accounted for 20.40% of the total; as such, common method bias was not a serious issue in this study.

Since the teacher–student relationship from teachers’ perspective was rated by 17 different head teachers, intraclass correlation coefficient (ICC) was calculated. One-way random effect model was chosen in the current study (Bliese, 1998). ICC = (MS_B_ – MS_W_)/[MS_B_ + (k – 1) ^∗^ MS_W_] (MS_B_ refers to the between-group mean square; MS_W_ refers to the within-group mean square; k refers to the arithmetic mean of group size). In the current study, MS_B_ = 0.78, MS_W_ = 0.21, *k* = 592/17 = 34.82; therefore, the teacher–student relationship from teachers’ perspective yielded an ICC of 0.07, indicating that the teacher-level factors’ systematical influence on the ratings of relationship was not serious in this study (Bliese, 1998).

Descriptive and correlation analyses of interpersonal trust, social adjustment, and teacher–student relationship were conducted using IBM SPSS 22.0. To test the indirect effect, structural equation modeling (SEM) followed by bootstrap analyses (bootstrap = 1000) (Preacher and Hayes, 2008) were conducted in Mplus 7.4. Several fit indices were used to evaluate model fit: χ^2^/*df* value ≤3, CFI and TLI values ≥0.90–0.94, RMSEA values ≤0.06–0.08, and SRMR values ≤0.06–0.08 indicated an adequate fit to the data (Hu and Bentler, 1998, 1999; Schermelleh-Engel et al., 2003). The full information likelihood method was used to deal with the missing data (Enders, 2010).

## Results

### Descriptive Statistics

Table 2 shows the descriptive statistics of all the variables in this study. The scores for interpersonal trust were the average of reliability, emotionality, and honesty (three aspects of interpersonal trust). The teacher–student relationship scores were the average of proximity, conflict, and reaction (three aspects of the teacher–student relationship). The scores for social adjustment were the average of social adjustment’s subordinate eight factors. On the whole, students reported medium-high levels of interpersonal trust, teacher–student relationship, and social adjustment. Head teachers reported higher levels of teacher–student relationship (*M* = 4.27 ± 0.47) than did students (*M* = 3.88 ± 0.54) (*t* = 14.82, *p* < 0.001, *d* = 0.77).

**TABLE 2:** Descriptive statistics of all variables.

	***M***	***SD***		***M***	***SD***
Interpersonal trust	3.90	0.67	Social adjustment	4.21	0.52
Reliability	4.14	0.66	Learning autonomy	4.21	0.66
Emotionality	3.67	0.76	Life independence	3.52	0.93
Honesty	3.89	0.85	Environment satisfaction	4.31	0.63
The teacher–student relationship from student’s perspectives	3.88	0.54	Friendly relationships	4.36	0.64
Proximity	3.83	0.70	Activity engagement	4.52	0.58
Conflict	4.22	0.75	Interpersonal coordination	4.47	0.54
Reaction	3.59	0.54	Social identification	3.96	0.76
The teacher–student relationship from teacher’s perspectives	4.27	0.47	Personal vitality	4.35	0.68
Proximity	4.19	0.71			
Conflict	4.58	0.58			
Reaction	4.04	0.43			

### Correlation Results

Table 3 reports the correlation results between interpersonal trust, social adjustment, teacher–student relationships from students’ and teachers’ perspectives, and demographic information. Interpersonal trust was positively correlated with social adjustment (*r* = 0.27, *p* < 0.001), the teacher–student relationship from students’ perspective (*r* = 0.10, *p* < 0.05), and the teacher–student relationship from teachers’ perspective (*r* = 0.36, *p* < 0.001). Social adjustment was positively correlated with the teacher–student relationship from students’ perspective (*r* = 0.54, *p* < 0.001), and the teacher–student relationship from teachers’ perspective (*r* = 0.18, *p* < 0.001). Social adjustment was positively correlated with gender (*r* = 0.12, *p* = 0.003), age (*r* = 0.13, *p* = 0.002), and grade (*r* = 0.14, *p* = 0.001).

**TABLE 3:** Correlation results of interpersonal trust, social adjustment, the teacher–student relationship, and demographic information.

	**1**	**2**	**3**	**4**	**5**	**6**	**7**	***M* ± *SD***
1. Interpersonal trust								3.90 ± 0.67
2. Social adjustment	0.27^∗∗∗^							4.21 ± 0.52
3. TSR-S	0.36^∗∗∗^	0.54^∗∗∗^						3.88 ± 0.54
4. TSR-T	0.10^∗^	0.18^∗∗∗^	0.20^∗∗∗^					4.27 ± 0.47
5. Gender	0.05	0.12^∗∗^	0.11^∗∗^	0.12^∗∗^				
6. Age	0.08^∗^	0.13^∗∗^	0.10^∗^	0.15^∗∗∗^	0.00			10.46 ± 0.92
7. Grade	0.07	0.14^∗∗^	0.09^∗^	0.24^∗∗∗^	0.04	0.80^***^		3.96 ± 0.78

TSR-S, the teacher–student relationship from students’ perspectives; TSR-T, the teacher–student relationship from teachers’ perspectives. *p < 0.05, **p < 0.01, ***p < 0.001.

### SEM and Indirect Effect Results

To examine the potential indirect effects of the teacher–student relationship between interpersonal trust and social adjustment, SEM followed by bootstrap analyses (bootstrap = 1000) (Preacher and Hayes, 2008) were conducted. First, one baseline model was tested, demonstrating the direct effect of children’s interpersonal trust on social adjustment. Next, three models were established separately to test the indirect roles of teacher–student relationship from the students’ perspective, the teachers’ perspective, and a combined perspective. Since the dependent variable social adjustment was positively correlated with gender, age, and grade, and the correlation between social adjustment and age (*r* = 0.13, *p* < 0.01) was lower than the correlation between social adjustment and grade (*r* = 0.14, *p* < 0.01); therefore, gender and grade were included in SEM as control variables.

**FIGURE 1:**
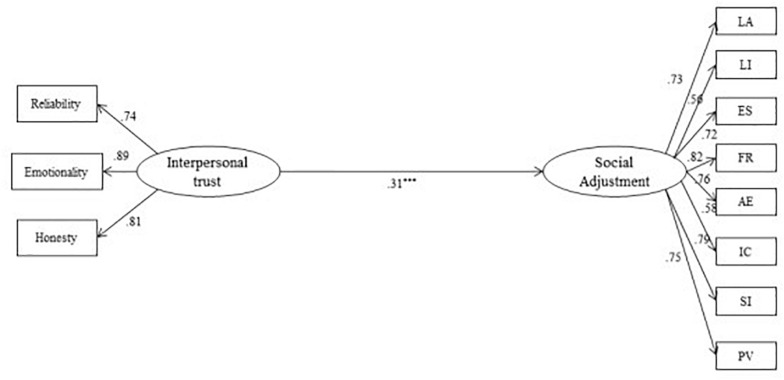
Standardized SEM of the Baseline Model (Model 1). LA, learning autonomy; LI, life independence; ES, environment satisfaction; FR, friendly relationships; AE, activity engagement; IC, interpersonal coordination; SI, social identification; PV, personal vitality. ***p < 0.001.

The SEM results for the baseline model are presented in Figure 1 (Model 1). The model achieved an acceptable fit: χ^2^/*df* = 3.14, CFI = 0.96, TLI = 0.95, RMSEA = 0.06 (90% CI of.051 to.070), SRMR = 0.05. The total R square for social adjustment was 0.12 (*p* < 0.001). The path of interpersonal trust on social adjustment was significant (β = 0.31, *p* < 0.001).

**FIGURE 2:**
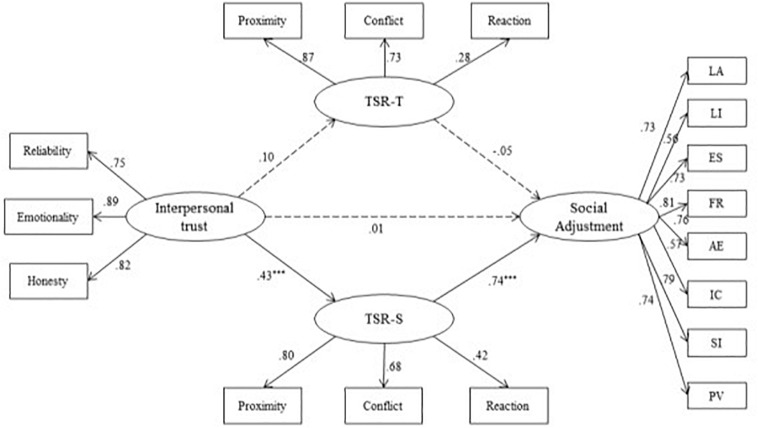
Standardized SEM of the Teacher-Student Relationships from Both Teachers’ Perspective and Students’ Perspective (Model 2). TSR-T, the teacher-student relationship from leather’s perspective; TSR-S, ihe teacher-student relationship from students perspective; LA, learning autonomy; LI, life independence; ES, environment satisfaction; FR, friendly relationships; AE, activity engagement; IC, interpersonal coordination; SI, social identification; PV, personal vitality. ***p < 0.001.

The SEM results for teacher–student relationships from both students’ and teachers’ perspectives are presented in Figure 2 (Model 2). The model fit was acceptable: χ^2^/*df* = 2.88, CFI = 0.94, TLI = 0.93, RMSEA = 0.06 (90% CI of 0.050 to 0.063), SRMR = 0.06. The total R square for social adjustment was 0.54 (*p* < 0.001). The direct path of interpersonal trust on social adjustment was not significant (β = 0.01, *p* > 0.05). The indirect path of the teacher–student relationship, from students’ perspectives, between interpersonal trust and social adjustment was 0.32 (*p* < 0.001, 95% CI of 0.23 to 0.43). The path between interpersonal trust and teacher–student relationship from teachers’ perspectives was not significant (β = 0.10, *p* > 0.05). The results indicate that the teacher–student relationship played a full indirect role between interpersonal trust and social adjustment from students’ perspectives, while it did not play a significant indirect role from teachers’ perspectives.

Next, we examined the gender differences in the mediation model. The interaction between interpersonal trust and gender had no significant influence on the teacher–student relationship from students’ perspectives (β = –0.00, *p* > 0.05); and the interaction between the teacher–student relationship from students’ perspectives and gender had no significant influence on social adjustment (β = 0.06, *p* > 0.05). Therefore, there was no gender difference in the mediation.

## Discussion

Previous studies have revealed the positive effect of children’s interpersonal trust on social adjustment (Betts and Rotenberg, 2007; Betts et al., 2013). However, the underlying psychological mechanism remains unclear. The current study assessed children’s interpersonal trust, social adjustment, and the teacher–student relationships from both students’ and teachers’ perspectives among grade 3–5 pupils in the Chinese context, demonstrating that the teacher–student relationship played an indirect role between children’s interpersonal trust and social adjustment from students’ perspectives rather than teachers’ perspectives, providing evidence that the teacher–student relationships from students’ and teachers’ perspectives have different effects on childhood social adjustment.

Whereas seldom previous empirical research explored the correlation of children’s interpersonal trust and teacher–student relationships, we first conducted the correlation analyses between the studied variables. Results showed that children’s interpersonal trust, the teacher–student relationship, and social adjustment were positively correlated with each other, which was consistent with our hypothesis. That is, for children with higher levels of interpersonal trust, they have better teacher–student relationships and social adjustment. This result was in line with the attachment theory and verified the previous findings (Bowlby, 1980; Pianta et al., 1995; Rotenberg et al., 2005a; Baker et al., 2008; Rotenberg, 2010; Liu et al., 2015; Ang et al., 2020; Ettekal and Shi, 2020). Therefore, hypothesis 1 failed to reject. Although the correlations between the studied variables were significant, some correlation coefficients were comparatively lower, that is, the correlation coefficient between interpersonal trust and the teacher–student relationship from teachers’ perspectives was lower than that between interpersonal trust and the teacher–student relationship from students’ perspectives; and the correlation coefficient between the teacher–student relationship from teachers’ perspectives and social adjustment was lower than that between the teacher–student relationship from students’ perspectives and social adjustment. These results suggested that the teacher–student relationship from students’ and teachers’ perspectives may play different roles between interpersonal trust and social adjustment.

Furthermore, previous studies mainly focused on the direct effect of children’s interpersonal trust on social adjustment (Betts and Rotenberg, 2007; Betts et al., 2013), while the current study has tested the full indirect effect of the teacher–student relationship between children’s interpersonal trust and social adjustment, explaining the psychological mechanism underlying the effect of children’s interpersonal trust on social adjustment. Compared to previous studies (Pianta et al., 1995; Hughes et al., 2012; Wang et al., 2013; Liu et al., 2015), the current study assessed the teacher–student relationships from both students’ and teachers’ perspectives. We tested the direct and indirect effects of the teacher–student relationship between interpersonal trust and social adjustment. The teacher–student relationship from students’ perspectives played a full indirect role between children’s interpersonal trust and social adjustment. The results revealed that teacher–student relationships from students’ and teachers’ perspectives predicted different outcomes, which was in line with the previous findings (Rey et al., 2007; Hughes, 2011), and the anchoring-and-adjustment heuristic (Gilovich et al., 2000; Epley, 2008).

Although one previous study on African American children revealed that teacher–student relationships from students’ and teachers’ perspectives predicted different outcomes (Rey et al., 2007), the current study found that, in the Chinese context, only the manner in which students perceived the teacher–student relationship mediated children’s interpersonal trust and social adjustment. This finding implies that students and teachers in the Chinese context evaluate and understand the teacher–student relationship differently, which may be explained by the culture of emphasizing respect for teachers (Liu et al., 2015) and large class sizes in China (Graue and Rauscher, 2009). In the current study, each head teacher was in charge of 42 students on average, and may not be able to pay adequate and equivalent attention to each student in her class, which leads to the different evaluation of the teacher–student relationship. Therefore, the teacher–student relationships differ between students’ and teachers’ perspectives, and play different indirect roles between children’s interpersonal trust and social adjustment in the Chinese context; hence hypothesis 2 failed to reject.

The current study has some contributions for this topic issue. The current study has demonstrated that the teacher-student relationship is influenced by children’s interpersonal trust, since no previous empirical study has focused on the effect of interpersonal trust on teacher-student relationship. In addition, the indirect role of teacher-student relationship between children’s interpersonal trust and social adjustment has been tested in the current study, which explains the psychological mechanism underlying the effect of children’s interpersonal trust on social adjustment. Also, the current study has explored the indirect role of the teacher-student relationships from both students’ and teachers’ perspectives in Chinese contexts, contributing to a culture-specific understanding of the teacher-student relationship’s roles in similar cultural contexts (e.g., Japan, South Korea, India) and schools with large class size (e.g., schools in Africa).

The current study has some practical implications. First, the indirect role of the teacher–student relationship between interpersonal trust and teacher–student relationship suggests that both teachers and parents should pay more attention toward the development of children’s interpersonal trust, which would lead to higher-quality teacher–student relationships. For example, building a harmonious family relationship and secure attachment will promote children’s interpersonal trust; and for those children with low levels of interpersonal trust, group counseling and sandplay therapy could be used to enhance interpersonal trust (Zhang et al., 2011). Second, our results show that students’ perception of the teacher–student relationship, rather than teachers’ perception, plays an indirect role between children’s interpersonal trust and social adjustment, suggesting that how students perceive the teacher–student relationship matters in childhood social adjustment. Teachers should exert more effort into building good relationships with students, and show more concern about how students perceive the relationship. Interventions such as teacher professional development and classroom-wide interventions can be put into practical use (Hughes et al., 2012). Third, teachers in large size classes may not give adequate and equivalent attention to each student, comparing to the small size classes. This finding suggests that educators should explore how to improve teachers’ concern for students in large size classes; and schools with large class sizes should work toward reducing these, so that the head teachers can show enough care to each student.

The current study has some limitations. First, we collected cross-sectional data only, which could not explain the causal relationship between children’s interpersonal trust and social adjustment. Subsequent studies could use different methods (e.g., longitudinal design) to explore the causal relationship. Second, we used convenient sampling in this research, namely, all participants were from one mid-level public primary school in Shanghai, China. Although we considered this school as a typical primary school in the Chinese context, future studies should replicate the findings in this study, by collecting data from different types of schools and different areas (including urban and rural). Last, we found evidence that students and teachers in the Chinese context may evaluate and understand the teacher–student relationship differently; however, comparative data (e.g., measuring the teacher–student relationship in other countries) was limited in the current study. Scholars may conduct cross-culture studies on the teacher–student relationship from different culture contexts.

## Conclusion

Our study revealed positive correlations between children’s interpersonal trust, the teacher–student relationship, and children’s social adjustment in the Chinese context. In addition, interpersonal trust indirectly influenced social adjustment through the teacher–student relationship from students’ perspectives, while the teacher–student relationship from teachers’ perspectives did not play an indirect role. The results indicated that students and teachers in the Chinese context evaluate and understand the teacher–student relationship differently, which may be explained by the culture of emphasizing respect for teachers and large class sizes in China.

## Data Availability Statement

The datasets presented in this study can be found in online repositories. The names of the repository/repositories and accession number(s) can be found in the article/Supplementary Material.

## Ethics Statement

The studies involving human participants were reviewed and approved by the Institutional Review Board, Department of Psychology, Renmin University of China. Written informed consent to participate in this study was provided by the participants’ legal guardian/next of kin. Written informed consent was obtained from the minor(s)’ legal guardian/next of kin for the publication of any potentially identifiable images or data included in this article.

## Author Contributions

YD, FL, and LC designed this study. YD collaborated in the final editing of manuscript and provided funding for the study. HW and ZL analyzed the data. HW wrote the initial draft of the manuscript and collaborated in the final editing manuscript. LC provided funding for the study. All authors contributed to the article and approved the submitted version.

## Conflict of Interest

The authors declare that the research was conducted in the absence of any commercial or financial relationships that could be construed as a potential conflict of interest.
